# An mHealth App to Increase Engagement in Mental Health Services for Depression and Anxiety: Protocol for a Pilot Randomized Controlled Trial

**DOI:** 10.2196/81645

**Published:** 2026-04-28

**Authors:** Aderonke Bamgbose Pederson

**Affiliations:** 1 Depression Clinical and Research Program Department of Psychiatry Massachusetts General Hospital Boston, MA United States

**Keywords:** stigma, mental health, interventions, illness, mental, services, mobile phone

## Abstract

**Background:**

Major depressive and anxiety disorders affect 61 million adults in the United States. People living with mental illness, including depression and anxiety, experience stigma associated with the illness and receiving treatment for their illness. Stigma refers to negative attitudes or beliefs about mental illness or negative behaviors directed toward persons with mental illness. Stigma is a leading and fundamental cause of health inequities. Contact interventions, which are premised on the idea that positive and voluntary contact with persons with mental illness can effectively reduce mental illness stigma, are aimed at reducing stigma and improving health outcomes. Video-based interventions improve knowledge, attitudes, and behavior in the short term, and there is a need for randomized controlled trials of indirect contact or video-based contact interventions to address stigma and engagement in mental health services.

**Objective:**

The aim is to assess the feasibility and acceptability and test the preliminary efficacy of a self-administered video-based mobile app in reducing mental illness stigma among Black adults with moderate to severe depression or anxiety.

**Methods:**

The intervention will involve short videos (6-12 minutes in length) of patients describing personal narratives of mental illness, treatment, and recovery and will be delivered over 4 weeks, with 2 booster sessions in weeks 6 and 12. Study participants (N=90) will be randomly assigned to 1 of 3 treatment arms: a standard video-based app (n=30), a video-based app modified with a concordant patient video (n=30), and a waitlist control (n=30).

**Results:**

We hypothesize the concordant and standard intervention group will have greater mental health service use, lower stigma, and lower mistrust compared to the waitlist arm. As a feasibility study, we will pilot the outcome analyses (to detect a signal and estimate direction of analysis). On the basis of the sample size of 90 (n=30 in each treated group and control group) and power at 72%, we will be able to detect a medium effect size of Cohen *d*=0.49 at α=.05 for comparing the intervention versus control proportions for any engagement in mental health care services.

**Conclusions:**

Given the low uptake of mental health services for people experiencing early signs and emerging symptoms of mental illness, efforts to increase early access to mental health services will be met with ongoing substantial barriers to mental health care use if stigma and medical mistrust are left unaddressed. Data from this pilot and feasibility randomized controlled trial will provide critical information about the feasibility and acceptability of this intervention and preliminary efficacy estimates. This study will fill a critical public health gap created by the stigma that leads to delayed diagnosis, delayed entry into mental health treatment, and increased morbidity and mortality related to mental illness.

**Trial Registration:**

ClinicalTrials.gov NCT06316804; https://clinicaltrials.gov/study/NCT06316804

**International Registered Report Identifier (IRRID):**

DERR1-10.2196/81645

## Introduction

### Background

Major depressive and anxiety disorders affect 61 million adults in the United States [1-3]. People living with mental illness, including depression and anxiety, experience stigma associated with the illness and receiving treatment for their illness [4,5]. Stigma, which refers to negative attitudes or beliefs about mental illness or negative behaviors directed toward persons with mental illness [6,7], is a leading and fundamental cause of health inequities [8-10]. The primary mechanisms through which stigma worsens mental health outcomes include delayed treatment seeking and limited access to care [4,11]. Contact interventions, which are premised on the idea that positive and voluntary contact with persons with mental illness can effectively reduce mental illness stigma [9,11,12], are aimed at reducing stigma and improving health outcomes. Past studies show face-to-face contact is superior to video-based contact; however, more recent randomized controlled trials (RCTs) show that video-based contact has a comparable effect in reducing stigma [4,7,13-16]. Video-based interventions improve knowledge, attitudes, and behavior in the short term, and there is a need for RCTs of indirect contact or video-based contact interventions to address stigma and engagement in mental health services [4,7,15,16].

Interventions to reduce mental illness stigma among Black adults remain understudied [4,17-19], and the role of mistrust is rarely examined [20]. There is a need for methodologically strong research to develop scalable interventions to reduce stigma and address medical mistrust, thus potentially increasing mental health service use [4,15,21]. Concomitant with these known gaps in the current literature, we developed iteratively and using a co-design user-centered design model, a mobile intervention platform to reduce stigma, address medical mistrust, and increase mental health service engagement among Black adults. In this study protocol manuscript, we present on the planned protocol for a novel randomized controlled intervention, testing the feasibility and acceptability of an antistigma mental health intervention delivered remotely on a digital mental health platform.

Several forms of stigma are prominent among persons with mental illness and contribute to increased psychological distress [8,9,11,22-24]: enacted stigma—the behavioral manifestations of negative attitudes about persons with mental illness, including stereotyping, social distancing, prejudice, and discrimination [25,26]; anticipated stigma—the expectation that one will be devalued for having mental illness and/or being subjected to stereotyping, prejudice, or discrimination if one’s mental illness were to become known by others [8,27-29]; and internalized stigma—endorsing the negative attitudes and beliefs as valid, thereby developing self-defacing beliefs about oneself [28,29]. Among Black adults, the content of mental health stigma includes beliefs that mental illness is attributed to a lack of faith in God, weakness, or lack of self-control [30-33]. However, current research studies approach Black adults as a homogenous group, and this assumption of homogeneity limits the efficacy of programs that seek to reduce mental illness stigma among Black adults in the United States [34-37]. Previous studies show that concordance in sociodemographic factors including race, ethnicity, and religiosity may improve the acceptability and efficacy of antistigma contact interventions [33].

Mobile mental health apps offer a mechanism to deliver interventions that can be modified in real time to align with user characteristics (such as race, ethnicity, or religious background) [38-42]. The largest systematic reviews and meta-analysis on stigma interventions to date have called for more culturally informed approaches to the design of stigma interventions [4,6,15,19,43]. In addition, concordance (eg, in race or ethnicity) between patients and providers is associated with higher trust, improved communication, and higher intent to adhere to health services [44-46]. Medical mistrust may be directed toward health professionals or health systems and is a known barrier to engagement in mental health services for the Black population [20,47,48]. Antistigma interventions that simultaneously address medical mistrust may have enhanced efficacy [20,49-51]. Previous studies show that having health professionals of shared racial, ethnic, or religious backgrounds would reduce their hesitancy toward accessing mental health services [46,52,53].

### Study Objective

The main study objective is to address mental health gaps around the role of stigma in reducing mental health service engagement by assessing a mobile app, codeveloped with end users. The user-centered design phase involved usability testing to refine intervention components to meet the needs and preferences of end users through an iterative, stepwise design process. Given the high stigma among underserved populations including Black adults with mental illness, screened participants in the user-centered design study reported a preference for key informant interviews over focus groups. A separate paper has been published on the app development of the intervention that is being tested and reported on in this protocol study manuscript. This paper describes the protocol for a 3-arm randomized controlled waitlist trial to assess the feasibility and preliminary efficacy of an antistigma mobile mental health app, which integrates components that address the mistrust of health systems, in reducing mental illness stigma among Black adults with moderate to severe depression or anxiety.

### Conceptual Framework

The conceptual model for stigma reduction is based on well-established sociological and psychological theories of behavior change. This model predicts that positive and voluntary contact with people living with mental illness can effectively reduce stigmatizing attitudes and stigmatizing behaviors [9,12,13]. On the basis of extensive research conducted by Corrigan et al [6], successful contact interventions have the following characteristics: targeted (ie, at a specific outcome, such as the use of mental health services); local (within a geographical region such as an urban setting); credible (ie, to the target group); continuously delivered (ie, not a single point of contact); and involves contact (closing the social gap between people with stigma and people living with mental illness through direct [in person] or indirect [video based or virtual] means).

Meta-analyses have consistently shown that both face-to-face and video-based contact with people living with mental illness can reduce stigma. In contrast to video-based contact, face-to-face interventions require intensive professional or peer facilitators, which significantly limits scaling, dissemination, and implementation goals [7,54,55]. The delivery of face-to-face stigma-reducing interventions can potentially be addressed by use of mobile mental health technology to deliver video-based interventions. Recent RCTs of interventions that optimize the core underpinnings of video contact show that video-based contact has similar efficacy as face-to-face contact [7,14,55-57].

## Methods

### Hypotheses

We hypothesize the concordant and standard intervention group will have greater mental health service use, lower stigma, and lower mistrust compared to the waitlist arm. We also hypothesize that among the two intervention groups, the concordant intervention group will have greater effects on the primary and secondary outcomes compared to the standard intervention group.

### Study Design and Setting

The study follows a single blind design among 2 experimental arms and 1 waitlist control arm. Participants are unaware of which intervention arm they are assigned among the two experimental arms. It was not feasible to fully blind the participants to the intervention versus waitlist arms, but we are able to blind participants to the specific content of the two intervention arms.

The intervention will involve short videos (6-12 minutes in length) of patients describing personal narratives of mental illness, treatment, and recovery and will be delivered over 4 weeks, with 2 booster sessions in weeks 6 and 12. Study participants (N=90) will be randomly assigned to 1 of 3 arms: a standard video-based app (n=30), a video-based app modified with a concordant patient video (n=30), and a waitlist control (n=30).

The study team used the SPIRIT (Standard Protocol Items: Recommendations for Interventional Trials) checklist in the development of the study protocol and conceptualization of the study and as study activities were initiated. We have included the page reference numbers for the SPIRIT checklist. The study team will follow the CONSORT (Consolidated Standards of Reporting Trails) checklist criteria throughout the study, including randomization scheme, study procedures, and planned analysis.

### Intervention Overview

#### Intervention Format

This study consists of a self-administered video-based stigma reduction app using an RCT design among Black adults (ages 18-45 years) from diverse ethnic backgrounds with moderate to severe depression and/or anxiety (9-item Patient Health Questionnaire ³10 or 7-item generalized anxiety disorder ³10) who have not accessed mental health services in the past year.

The intervention videos involve 6- to 12-minute videos, offering narratives on treatment and recovery by people living with mental illness from diverse backgrounds including narratives of depression illness and recovery [58,59]. In the adapted (concordant) intervention arm, participants will be offered video content from someone with the same or similar sociodemographic background based on their reported characteristics at screening [60]. The standard (discordant) intervention arm will involve video content from someone from a different sociodemographic background. The 2 intervention conditions will be delivered on a secure HIPAA (Health Insurance Portability and Accountability Act)-compliant platform. Content will be available in English at an eighth-grade reading level [61,62]. New sessions will be prompted each week based on the experimental condition arm, and usage data will be collected. The study will include individuals from various age groups, education and income levels, and gender identities. Study participants will be randomly assigned to 1 of 3 arms: a standard video-based app (n=30), a video-based app modified with a concordant individual (n=30), and a waitlist control (n=30).

#### Setting, Participants, and Eligibility Criteria

Recruitment will take place through the Massachusetts General Hospital patient registry, online social media advertising, and in partnership with community-based organizations. All participants will be prescreened via a phone call and/or an online form. All participants will complete an orientation call prior to randomization. To ensure representativeness of the sample, we will use a stratified random sampling of study participants to include equitable numbers of genders, migrant status, and ethnic origin (African Americans, African immigrants, Afro-Caribbean immigrants, and Afro-Latina). The participant eligibility criteria are shown in Textbox 1.

**Participant eligibility criteria.**

**Inclusion criteria**

- Black adults with moderate to severe depression (9-item Patient Health Questionnaire ³10) or anxiety (7-item generalized anxiety disorder ³10) who are not receiving routine mental health care
- Aged 18 to 45 years at time of screening. This age range was chosen due to variability in technology across age groups, reducing the heterogeneity of technology use among participants.
- English speaking
- Own a smartphone and have internet access

**Exclusion criteria**

- Visual, hearing, voice, or motor impairments that would prevent engagement in study procedures
- Diagnosis of psychotic disorder or severe suicidality for which participation would be inappropriate

Language variance and lack of access to a smartphone would prevent engagement in study procedures and impact feasibility assessment.

#### Procedure

At the initial visit, we will obtain baseline measurements of enacted, anticipated, and internalized stigma [8]. The self-administered intervention will be delivered over 4 weeks based on the experimental condition, and participants will each receive a standard video-based intervention or a concordant video-based intervention. Each week, participants will be prompted with a notification (via SMS text message and/or email) to access that week’s intervention components. The content of booster sessions in weeks 6 and 12 will be based on the experimental arm and will contain repeat sessions used in each experimental arm. The waitlist arm will receive the concordant video condition after 12 months. At the orientation visit, for all participants, guidance on obtaining emergent or urgent services is provided, including reaching out to the study team (which includes a social worker, resource specialist, and psychiatrist) at any time.

#### Recruitment

Patients who present for general medical care at the selected Mass General Brigham community clinic partners are routinely given screening tests (9-item Patient Health Questionnaire and 7-item generalized anxiety disorder). The community advisory board will support recruitment efforts. Flyers and announcements will be available in clinics and shared with community partners. Any participant who meets the entry criteria on screening tests and who may have declined mental health services will be offered the stigma intervention program. If a patient agrees to participate, a trained research assistant will contact the patient. In addition, using the electronic medical records, patients who meet the criteria within the last 12 months and have not received mental health services in the past year will be invited to participate in the eligibility screening. The waitlist arm will receive holiday cards and text reminders to complete assessments. Incentives to participate in the stigma intervention include offering a low intensity, nonjudgmental, and confidential option to patients who have screened positive for depression or anxiety but who are not receiving routine mental health care. To manage attrition in app use, which is common, we will send out weekly reminders using multiple methods such as email, text, and phone call reminders for participants missing study activities or assessments.

#### Assessments

Study recruitment and retention rates will be assessed to determine the feasibility of study procedures (Table 1). The feasibility outcomes will be measured based on acceptability of the study through measures of usability: System Usability Scale (SUS) and usefulness, satisfaction, and ease of use (USE) scales [63,64]. We will also document adherence based on time spent on the intervention program and app use data to assess the completion of video activities. Assessments will be completed using standardized measures (Table 1).

**Table 1:** Intervention feasibility trial process and outcomes.

Domain, construct, and measure				Baseline		Each week		After the booster		Follow-up at 6-12 months
**Feasibility outcomes**										
	**Acceptability**									
		USE^a^ and SUS^b^			✓		✓		✓	
	**Retention to intervention**									
		Completed assessments			✓		✓		✓	
	**Adherence to intervention**									
		App metrics			✓		✓		✓	
**Primary outcomes**										
	**Demographic**									
		Survey	✓							
	**Any engagement with mental health services**									
		EMR^c^	✓						✓	
**Secondary outcomes**										
	**Sigma scores**									
		RIBS^d^ and ISMI^e^	✓		✓		✓		✓	
	**Help-seeking behavior**									
		GHSQ^f^	✓		✓		✓		✓	
	**Medical mistrust scores**									
		GBMMS^g^	✓		✓		✓		✓	
**Targets**										
	**Sigma and medical mistrusts**									
		RIBS, ISMI, and GBMMS	✓		✓		✓		✓	

^a^USE: usefulness, satisfaction, and ease of use.

^b^SUS: System Usability Scale.

^c^EMR: electronic medical record.

^d^RIBS: Reported and Intended Behavior Scale.

^e^ISMI: Internalized Stigma of Mental Illness.

^f^GHSQ: General Help-Seeking Questionnaire.

^g^GBMMS: Group-Based Medical Mistrust Scale.

#### Data Analysis and Power Calculation

As a feasibility study, we will pilot the outcome analyses (to detect a signal and estimate direction of analysis), recognizing that the trial may be underpowered. On the basis of the sample size of 90 (n=30 in each treated group and control group) and power at 72%, we will be able to detect a medium effect size of Cohen *d*=0.49 at α=.05 comparing the intervention versus control proportions for any engagement in mental health care services.

For the continuous outcomes (such as Group-Based Medical Mistrust Scale), we will use generalized linear mixed models with a gamma distribution for continuous outcomes. If variables are not normally distributed, we will explore a transformation. For binary outcomes, we will use Poisson or negative binomial regression depending on the variance.

### Randomization and Blinding

#### Randomization Scheme

**Figure 1:**
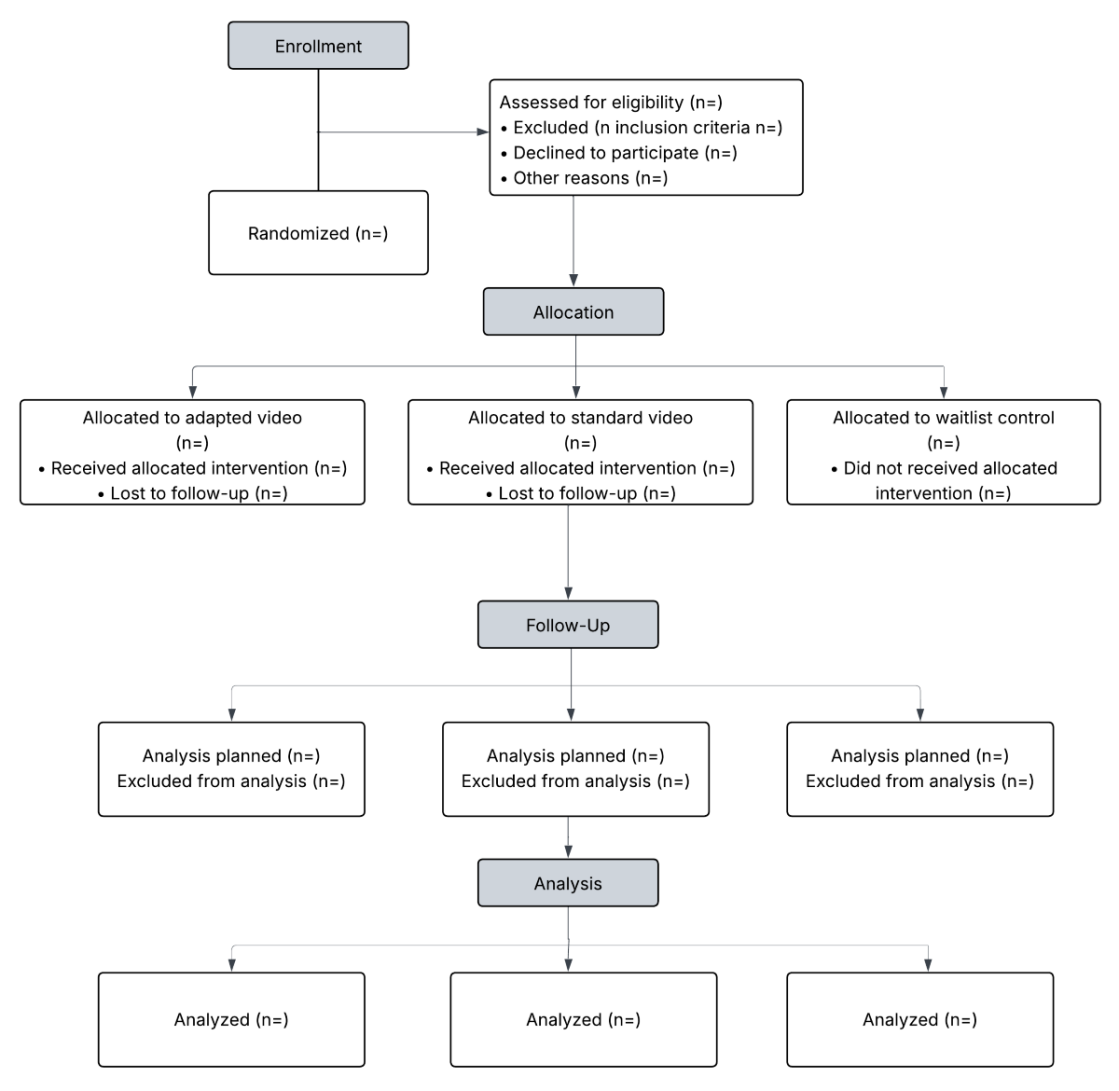
CONSORT (Consolidated Standards of Reporting Trails) diagram.

Participants will be randomized to 1 of 3 arms: a standard video contact arm, a concordant video contact arm, and a waitlist control arm as outlined in our CONSORT diagram (Figure 1). Assignment to the 3 arms in a 1:1:1 allocation ratio will be determined centrally by the principal investigator according to a computer-generated random schedule (ie, in Stata [StataCorp]). There is no practical way to fully blind the study participants to treatment assignment versus waitlist and accomplish the objectives of the study. To avoid bias among participants receiving the concordant or standard video assignment, we will conceal treatment arm allocation. We are testing the salience of concordance across sociodemographic characteristics; unblinding would prevent unbiased testing of the role of concordance. The study participants will be blinded to the two intervention arms and will be unaware which intervention arm they were assigned throughout the study. The research study staff and biostatistician (who will be conducting the outcome assessments and analysis, respectively) will be blinded to treatment assignment status. The participants assigned to the waitlist will receive access to the concordant video contact intervention after they have completed their 12-month study assessment. To avert chance imbalances by ethnicity and sex, we will stratify randomization by ethnicity (African American, Afro-Caribbean or Afro-Latino, and African immigrant) and sex. The regression models for the primary analysis include the stratification variables as covariates to ensure the estimated standard errors are accurate. We hypothesize that the concordant video arm will be superior to the standard video arm. The standard video arm is needed to test our hypothesis and understand the mechanistic effect of each therapeutic target.

Reliability and validity data for the study assessments are shown in Table 2 [65-68].

**Table 2:** Reliability and validity data for the study assessments.

Variables	Reliability and validity	Estimated administration time
4-item Reported and Intended Behavior Scale (Evans-Lacko et al [66], 2011)	Reliability: overall test-retest reliability was 0.75, and internal consistency, based on Cronbach α among items 5-8, was 0.85.	2 minutes
29-item Internalized Stigma of Mental Illness (Boyd et al [65], 2014)	Reliability: the original ISMIa reported test-retest reliability of 0.92	4-6 minutes
12-item Group-Based Medical Mistrust Scale (Shelton et al [67], 2010)	Reliability: Cronbach α for the full measure in previous studies (α=0.87-0.88). Validity: the total GBMMSb score and its 3 subscales were positively correlated with avoidance of health care (total score: *P*<.001; r=0.344).	3-5 minutes
20-item General Help-Seeking Questionnaire (Ibrahim et al [68], 2019)	Reliability: Cronbach α for the full measure in previous studies (α=0.91). Validity: the perceived quality of previous mental health care was positively related to intentions to seek help from a mental health professional for personal and emotional problems (rs55=0.51, *P*<.001) and suicidal thoughts (rs54=0.57, *P*<.001).	3-5 minutes

^a^ISMI: Internalized Stigma of Mental Illness.

^b^GBMMS: Group-Based Medical Mistrust Scale.

#### Missing Data

Data missing at follow-up could bias study findings. We plan to record the reason for dropout. When dropout occurs completely at random, the statistical analyses stated will provide appropriate statistical inferences. The multiple imputation approach will be conducted for follow-up data that are missing at random.

#### Planned and Interim Analysis

We hypothesize that the contact-based intervention arm that incorporates an approach using concordance will reduce stigma and lower medical mistrust, improving engagement in mental health services. Feasibility will be assessed by meeting our recruitment goals (90 participants), obtaining 80% of follow-up self-report data, and having a dropout rate of <20% in one or more treatment conditions, which is in line with previous mobile mental health intervention RCTs [69] and which would indicate the treatment format for future research. Recruitment and retention rates, acceptability, adherence to study, and treatment will be reported as percentages. The average USE and SUS scores will be reported.

We will assess the moderating role of sociodemographic factors (religiosity, migrant status, and socioeconomic status) by controlling for these variables in our pre-post assessments.

### Ethical Considerations

This study has been approved by the institutional review board (IRB) of Massachusetts General Brigham (2022P000580). Full informed consent will be obtained by the principal investigator (a licensed physician) prior to study activities. A data safety monitoring board is not needed for a minimal risk intervention (IRB determination). This study was registered on ClinicalTrials.gov (NCT06316804).

Protocol deviations are documented and reported to the IRB and sponsor based on Massachusetts General Brigham IRB policy. Minor deviations are reported at continuing review based on the designated timeline by the IRB. Any major deviations are reported at time of identifying the deviation based on the IRB policy. All participants will complete a virtual orientation visit (prior to baseline assessment). At this visit, waitlist participants and intervention participants are provided guidance on obtaining emergent or urgent referral services, including reaching out to the study team (which includes a social worker, resource specialist, and psychiatrist) at any time throughout the study period.

All participants complete full informed consent prior to study activities. The study is voluntary and participants may discontinue participation at any time. The study team will ensure participants are able to safely discontinue participation. Only deidentified information is used for analysis and publication purposes. To help with retention, starting at US $20 for the initial baseline assessment, we will offer a graded compensation schedule with US $5 to US $10 increments for subsequent assessments completed.

### Data Dissemination and Data Availability

The study results will be presented at conferences and in relevant journals. After data collection and initial analysis is completed and published, data will be managed based on sponsor (National Institutes of Health) data management and privacy policy. Deidentified data will be available in accordance with the National Institute of Mental Health Data Archive policy and timeline.

## Results

### Study Status

The study was funded on August 16, 2022. Initial activities involved app design. Following app design, clinical trial activities will be initiated. As of submission of the manuscript, 75 of 90 participants were successfully recruited. Study results will be published in March 2027.

### Study Outcomes

Our study will assess our ability to recruit and retain study participants and measure usability, satisfaction, and adherence. The primary outcome will be mental health service use, assessed 4 weeks after the intervention and at 3 months after the booster sessions and again at 6 and 12 months, following booster sessions. Secondary outcomes will include anticipated, enacted, and internalized stigma; medical mistrust; and help-seeking behavior. The primary outcome is defined as any engagement with mental health services: this includes intake appointments, follow-up appointments, or scheduled appointments for mental health with a psychiatrist, physician or clinician, clinical psychologist, therapist, or self-report mental health service use (verified by electronic health records). At baseline, participants sign a release of information (ROI) form to allow the study team to obtain their medical records from their designated health system. The ROI form expires at 6 months, and a new ROI will be obtained to ensure the study team is able to retrospectively review use of mental health services at study completion at 12 months.

### Data Collection and Outcomes

Data collection will be done through a secure Research Electronic Data Capture (REDCap; Vanderbilt University) database. Our app platform allows us to track and collect data on app engagement and usage metrics.

The secondary outcomes will be anticipated, enacted, and internalized stigma; medical mistrust; and self-reported help-seeking behavior. Feasibility outcomes will be assessed, including acceptability (USE and SUS), adherence (use metrics), and retention in the study (online assessments completed). We define a priori targets for the SUS and USE scales as usability scores >70, indicating good to excellent usability and mean scores on the USE scales (comparing the two experimental arms). We will measure study adherence based on the number of app logins and target at least one video session and assessment completed.

## Discussion

### Expected Findings

Black adults are known to have lower use of mental health services. In light of the increasing use of mobile devices, a self-administered antistigma mobile app has the potential to enhance accessibility and scalability of traditionally difficult to access stigma reduction tools. This manuscript describes the study protocol for an ongoing RCT to assess 2 intervention arms (a concordant and standard video-based stigma-reducing mobile app) compared with a waitlist control group. We hypothesize that individuals assigned to the intervention groups will be more likely to choose to engage in mental health services and report decreased stigma and decreased mistrust compared with the waitlist control group. Additional secondary outcomes will allow an estimate of the extent to which changes in anticipated or enacted or internalized stigma and medical mistrust mediate the intervention’s effect on the primary and secondary outcomes. Mediation analysis will be conducted to assess whether stigma (Reported and Intended Behavior Scale and Internalized Stigma of Mental Illness) and medical mistrust (Group-Based Medical Mistrust Scale) are potential mediators in the pathway between engagement in mental health care and the intervention [65,66,68,70,71]. Causal mediation analyses will be conducted to assess the relationships. The direct and indirect effects from the causal mediation analyses will be estimated. Percent of total mediation effect and 95% CI from each causal mediation analysis will be provided. We will use the SAS (SAS Institute) procedure CAUSALMED based on methods developed by Valeri and Vanderweele [72].

### Study Status

This is an ongoing randomized controlled clinical trial. Recruitment of participants started in July 2024 and is expected to be completed in July 2026 for the 1-year surveillance follow-up. The study is currently under protocol version 3 (approved in December 2024).

### Future Directions

Given the low uptake of mental health services for people experiencing early signs and emerging symptoms of mental illness, efforts to increase early access to mental health services will be met with ongoing significant barriers to mental health care use if stigma and medical mistrust are left unaddressed. Data from this pilot and feasibility randomized trial will provide critical information about the feasibility and acceptability of this intervention and preliminary efficacy estimates.

### Limitations

The sample size is insufficient to definitively test the effectiveness of the intervention for the primary outcomes. However, prior to making large investments in this line of study, delineating mechanistic targets and identifying key outcome assessments and measures will be critical to informing future investment in fully powered RCTs and implementation studies. The use of mobile technology varies across age groups. To address this limitation, we have chosen to include a narrow age group (aged 18-45 years) while we test the feasibility and acceptability of the mobile app.

### Conclusions

Despite the limitations, the next step is to further refine the intervention (based on this clinical trial) for testing in a fully powered randomized controlled effectiveness trial. Future studies will be adequately informed on the refined approach in testing the effectiveness and implementation of this mobile app and other similar mobile health apps across disease conditions and across populations at risk of worsening of disease due to treatment hesitancy and high stigma. While the study is underpowered to test effectiveness, this study will fill a critical public health gap created by the stigma that leads to delayed diagnosis, delayed entry into mental health treatment, and increased morbidity and mortality related to mental illness.

## Abbreviations

CONSORT: Consolidated Standards of Reporting Trails
HIPAA: Health Insurance Portability and Accountability Act
IRB: institutional review board
RCT: randomized controlled trial
ROI: release of information
SPIRIT: Standard Protocol Items: Recommendations for Interventional Trials
SUS: System Usability Scale
USE: usefulness, satisfaction, and ease of use

## References

[1] Major depression. National Institute of Mental Health.

[2] What is depression?. Anxiety & Depression Association of America.

[3] Dattani S, Rodés-Guirao L, Ritchie H, Roser M (2023). Mental health. Our World in Data.

[4] Thornicroft G, Mehta N, Clement S, Evans-Lacko S, Doherty M, Rose D, Koschorke M, Shidhaye R, O'Reilly C, Henderson C (2016). Evidence for effective interventions to reduce mental-health-related stigma and discrimination. The Lancet.

[5] McCullock SP, Scrivano RM (2023). The effectiveness of mental illness stigma-reduction interventions: a systematic meta-review of meta-analyses. Clin Psychol Rev.

[6] Corrigan PW, Morris SB, Michaels PJ, Rafacz JD, Rüsch N (2012). Challenging the public stigma of mental illness: a meta-analysis of outcome studies. Psychiatr Serv.

[7] Amsalem D, Jankowski SE, Yanos P, Yang LH, Markowitz JC, Rogers RT, Stroup TS, Dixon LB, Pope LG (2024). Randomized controlled trial of a brief video intervention to reduce self-stigma of mental illness. J Clin Psychiatry.

[8] Fox AB, Earnshaw VA, Taverna EC, Vogt D (2018). Conceptualizing and measuring mental illness stigma: the mental illness stigma framework and critical review of measures. Stigma Health.

[9] Kolb K, Liu J, Jackman K (2023). Stigma towards patients with mental illness: an online survey of United States nurses. Int J Ment Health Nurs.

[10] Hatzenbuehler ML, Phelan JC, Link BG (2013). Stigma as a fundamental cause of population health inequalities. Am J Public Health.

[11] Adu J, Oudshoorn A, Anderson K, Marshall CA, Stuart H (2022). Social contact: next steps in an effective strategy to mitigate the stigma of mental illness. Issues Ment Health Nurs.

[12] Cerully JL, Collins RL, Wong E, Seelam R, Yu J (2018). Differential response to contact-based stigma reduction programs: perceived quality and personal experience matter. Psychiatry Res.

[13] Koike S, Yamaguchi S, Ojio Y, Ohta K, Shimada T, Watanabe K, Thornicroft G, Ando S (2018). A randomised controlled trial of repeated filmed social contact on reducing mental illness-related stigma in young adults. Epidemiol Psychiatr Sci.

[14] Vinson ES, Abdullah T, Brown TL (2016). Mental illness stigma intervention in African Americans: examining two delivery methods. J Nerv Ment Dis.

[15] Mehta N, Clement S, Marcus E, Stona AC, Bezborodovs N, Evans-Lacko S, Palacios J, Docherty M, Barley E, Rose D, Koschorke M, Shidhaye R, Henderson C, Thornicroft G (2015). Evidence for effective interventions to reduce mental health-related stigma and discrimination in the medium and long term: systematic review. Br J Psychiatry.

[16] Makhmud A, Thornicroft G, Gronholm PC (2022). Indirect social contact interventions to reduce mental health-related stigma in low- and middle-income countries: systematic review. Epidemiol Psychiatr Sci.

[17] Yamaguchi S, Wu SI, Biswas M, Yate M, Aoki Y, Barley EA, Thornicroft G (2013). Effects of short-term interventions to reduce mental health-related stigma in university or college students: a systematic review. J Nerv Ment Dis.

[18] Subica AM, Link BG (2026). Mental illness stigma in Black, Latina/o, and Asian Americans. J Racial Ethn Health Disparities.

[19] Misra S, Jackson VW, Chong J, Choe K, Tay C, Wong J, Yang LH (2021). Systematic review of cultural aspects of stigma and mental illness among racial and ethnic minority groups in the United States: implications for interventions. Am J Community Psychol.

[20] Powell W, Richmond J, Mohottige D, Yen I, Joslyn A, Corbie-Smith G (2019). Medical mistrust, racism, and delays in preventive health screening among African-American men. Behav Med.

[21] Rodríguez-Rivas ME, Cangas AJ, Martin A, Romo J, Pérez JC, Valdebenito S, Cariola L, Onetto J, Hernández B, Ceric F, Cea P, Corrigan P (2024). Reducing stigma toward people with serious mental illness through a virtual reality intervention: a randomized controlled trial. Games Health J.

[22] Quinn DM, Earnshaw VA (2013). Concealable stigmatized identities and psychological well-being. Soc Personal Psychol Compass.

[23] Ritsher JB, Phelan JC (2004). Internalized stigma predicts erosion of morale among psychiatric outpatients. Psychiatry Res.

[24] Goetz CJ, Mushquash CJ, Maranzan KA (2023). An integrative review of barriers and facilitators associated with mental health help seeking among indigenous populations. Psychiatr Serv.

[25] Link BG, Cullen FT, Frank J, Wozniak JF (1987). The social rejection of former mental patients: understanding why labels matter. Am J Sociol.

[26] Tan KK, Treharne GJ, Ellis SJ, Schmidt JM, Veale JF (2021). Enacted stigma experiences and protective factors are strongly associated with mental health outcomes of transgender people in Aotearoa/New Zealand. Int J Transgend Health.

[27] Quinn DM, Earnshaw VA (2011). Understanding concealable stigmatized identities: the role of identity in psychological, physical, and behavioral outcomes. Soc Issues Policy Rev.

[28] Link BG, Cullen FT, Struening E, Shrout PE, Dohrenwend BP (1989). A modified labeling theory approach to mental disorders: an empirical assessment. Am Sociol Rev.

[29] O'Donnell AT, Foran AM (2024). The link between anticipated and internalized stigma and depression: a systematic review. Soc Sci Med.

[30] Pederson AB, Hawkins D, Lartey L (2026). Descriptive analysis of social determinant of mental health factors in an ethnically diverse Black adult population. medRxiv. Preprint posted online on October 29, 2021.

[31] Bamgbose Pederson A, Waldron EM, Fokuo JK (2022). Perspectives of Black immigrant women on mental health: the role of stigma. Womens Health Rep (New Rochelle).

[32] Pederson AB, Konadu Fokuo J, Thornicroft G, Bamgbose O, Ogunnubi OP, Ogunsola K, Oshodi YO (2023). Perspectives of university health care students on mental health stigma in Nigeria: qualitative analysis. Transcult Psychiatry.

[33] Pederson AB, Earnshaw VA, Lewis-Fernández R, Hawkins D, Mangale DI, Tsai AC, Thornicroft G (2023). Religiosity and stigmatization related to mental illness among African Americans and Black immigrants: cross-sectional observational study and moderation analysis. J Nerv Ment Dis.

[34] Williams DR, González HM, Neighbors H, Nesse R, Abelson JM, Sweetman J, Jackson JS (2007). Prevalence and distribution of major depressive disorder in African Americans, Caribbean Blacks, and non-Hispanic Whites: results from the National Survey of American Life. Arch Gen Psychiatry.

[35] Pederson AB, Hawkins D, Lartey L (2022). Differences in psychosocial factors of mental health in an ethnically diverse Black adult population. J Public Health Policy.

[36] Bijou C, Colen CG (2022). Shades of health: skin color, ethnicity, and mental health among Black Americans. Soc Sci Med.

[37] Amuta-Jimenez AO, Jacobs W, Smith G (2020). Health disparities and the heterogeneity of Blacks/African Americans in the United States: why should we care?. Health Promot Pract.

[38] Yeo G, Reich SM, Liaw NA, Chia EY (2024). The effect of digital mental health literacy interventions on mental health: systematic review and meta-analysis. J Med Internet Res.

[39] Timakum T, Xie Q, Song M (2022). Analysis of e-mental health research: mapping the relationship between information technology and mental healthcare. BMC Psychiatry.

[40] Taylor C, Graham AK, Flatt RE, Waldherr K, Fitzsimmons-Craft EE (2021). Current state of scientific evidence on internet-based interventions for the treatment of depression, anxiety, eating disorders and substance abuse: an overview of systematic reviews and meta-analyses. Eur J Public Health.

[41] Almuqrin A, Hammoud R, Terbagou I, Tognin S, Mechelli A (2025). Smartphone apps for mental health: systematic review of the literature and five recommendations for clinical translation. BMJ Open.

[42] Rubeis G (2021). E-mental health applications for depression: an evidence-based ethical analysis. Eur Arch Psychiatry Clin Neurosci.

[43] Mpompola T, Zartaloudi A, Mantoudi A, Mantzorou M, Apostolara P, Adamakidou T (2026). Attitudes of healthcare professionals working in primary healthcare towards people with mental illness: a systematic review. Adv Exp Med Biol.

[44] Greenwood BN, Hardeman RR, Huang L, Sojourner A (2020). Physician-patient racial concordance and disparities in birthing mortality for newborns. Proc Natl Acad Sci U S A.

[45] Alsan M, Garrick O, Graziani G (2019). Does diversity matter for health? Experimental evidence from Oakland. Am Econ Rev.

[46] Jackson JL, Apilado KP, Koehlmoos TP (2025). A review of reviews assessing patient-provider racial and ethnic concordance in mental health. J Racial Ethn Health Disparities.

[47] Henderson RC, Williams P, Gabbidon J, Farrelly S, Schauman O, Hatch S, Thornicroft G, Bhugra D, Clement S (2015). Mistrust of mental health services: ethnicity, hospital admission and unfair treatment. Epidemiol Psychiatr Sci.

[48] Colvin K, Potts W, Heinlein E, Himelhoch S (2024). Prevalence and predictors of medical mistrust among African Americans with serious mental illness receiving care in an urban setting. Community Ment Health J.

[49] Holland S, Stocks D (2017). Trust and its role in the medical encounter. Health Care Anal.

[50] Smirnoff M, Wilets I, Ragin DF, Adams R, Holohan J, Rhodes R, Winkel G, Ricci EM, Clesca C, Richardson LD (2018). A paradigm for understanding trust and mistrust in medical research: the Community VOICES study. AJOB Empir Bioeth.

[51] Pederson AB, McLaughlin C, Hawkins D, Jain FA, Anglin DM, Yeung A, Tsai AC (2025). Medical mistrust and willingness to use mental health services among a cohort of Black adults. Psychiatr Serv.

[52] Sullivan LS (2020). Trust, risk, and race in American medicine. Hastings Cent Rep.

[53] Lewis JE, Pride LC, Luk HG, Oyejide K, Wilson IM, Tawiah WE, Watkins CM, Lee WC (2024). Aligning our actions with our words: a systematic review of gender and racial diversity in surgical subspecialties. J Med Access.

[54] Yanos PT, Lucksted A, Drapalski AL, Roe D, Lysaker P (2015). Interventions targeting mental health self-stigma: a review and comparison. Psychiatr Rehabil J.

[55] Amsalem D, Jankowski SE, Pagdon S, Smith S, Yang LH, Valeri L, Markowitz JC, Lewis-Fernández R, Dixon LB (2024). "It's tough to be a Black man with schizophrenia": randomized controlled trial of a brief video intervention to reduce public stigma. Schizophr Bull.

[56] Heim E, Henderson C, Kohrt BA, Koschorke M, Milenova M, Thornicroft G (2019). Reducing mental health-related stigma among medical and nursing students in low- and middle-income countries: a systematic review. Epidemiol Psychiatr Sci.

[57] Amsalem D, Markowitz JC, Jankowski SE, Yang LH, Valeri L, Lieff SA, Neria Y, Dixon LB (2021). Sustained effect of a brief video in reducing public stigma toward individuals with psychosis: a randomized controlled trial of young adults. Am J Psychiatry.

[58] Chris Hubbard: tackling mental health stigma. NAMI YouTube page.

[59] (2021). From self-termination watch to recovery: "this isn’t me just being broken". NAMI YouTube page.

[60] Mental health stigma. caliclown5 YouTube page.

[61] Bakken S, Grullon-Figueroa L, Izquierdo R, Lee NJ, Morin P, Palmas W, Teresi J, Weinstock RS, Shea S, Starren J (2006). Development, validation, and use of English and Spanish versions of the telemedicine satisfaction and usefulness questionnaire. J Am Med Inform Assoc.

[62] Brewer CF (2026). Evaluating the readability of clinical trial content for patients on pharmaceutical company websites. Pharmaceut Med.

[63] Brooke J, Jordan PW, Thomas B, McClelland IL, Weerdmeester B (1996). SUS: a 'quick and dirty' usability scale. Usability Evaluation in Industry.

[64] Gao M, Kortum P, Oswald F (2018). Psychometric evaluation of the USE (Usefulness, Satisfaction, and Ease of use) Questionnaire for reliability and validity. Proc Hum Factors Ergon Soc Annu Meet.

[65] Boyd JE, Adler EP, Otilingam PG, Peters T (2014). Internalized Stigma of Mental Illness (ISMI) scale: a multinational review. Compr Psychiatry.

[66] Evans-Lacko S, Rose D, Little K, Flach C, Rhydderch D, Henderson C, Thornicroft G (2011). Development and psychometric properties of the reported and intended behaviour scale (RIBS): a stigma-related behaviour measure. Epidemiol Psychiatr Sci.

[67] Shelton RC, Winkel G, Davis SN, Roberts N, Valdimarsdottir H, Hall SJ, Thompson HS (2010). Validation of the Group-Based Medical Mistrust Scale among urban Black men. J Gen Intern Med.

[68] Ibrahim N, Amit N, Shahar S, Wee LH, Ismail R, Khairuddin R, Siau CS, Safien AM (2019). Do depression literacy, mental illness beliefs and stigma influence mental health help-seeking attitude? A cross-sectional study of secondary school and university students from B40 households in Malaysia. BMC Public Health.

[69] Linardon J, Fuller-Tyszkiewicz M (2020). Attrition and adherence in smartphone-delivered interventions for mental health problems: a systematic and meta-analytic review. J Consult Clin Psychol.

[70] Clement S, Brohan E, Jeffery D, Henderson C, Hatch SL, Thornicroft G (2012). Development and psychometric properties the Barriers to Access to Care Evaluation scale (BACE) related to people with mental ill health. BMC Psychiatry.

[71] Thompson HS, Valdimarsdottir HB, Winkel G, Jandorf L, Redd W (2004). The Group-Based Medical Mistrust Scale: psychometric properties and association with breast cancer screening. Prev Med.

[72] Valeri L, Vanderweele TJ (2013). Mediation analysis allowing for exposure-mediator interactions and causal interpretation: theoretical assumptions and implementation with SAS and SPSS macros. Psychol Methods.

